# A Gestalt therapeutic approach to supporting people presenting with bulimia

**DOI:** 10.1186/2050-2974-3-S1-P5

**Published:** 2015-11-23

**Authors:** Isa Pfluger

**Affiliations:** 1 Isis - The Eating Issues Centre, Australia

## 

Gestalt therapy's dialogic, field-sensitive and experiential approach offers unique opportunities to address some of the core challenges of people presenting with Bulimia (BN). Employing an attitude of curiosity and exploration, Gestalt therapists are able to engage clients at various stages of change, and the approach is well suited to working with the ambivalence often encountered early in treatment.

BN can be conceptualised as a creative adjustment, as the best option available at the time given current resources, to manage overwhelming affect and unacceptable feelings and needs. Working experientially facilitates an embodied awareness that enables disowned feelings and needs to emerge within sessions. With sufficient relational support and sensitive attendance to the emergence of shame, affect can be co-regulated, and feelings, which were too hard to bear in isolation, can be experienced, named and shared.

Over time clients' capacity to remain in contact with these previously disowned feelings and needs increases. First steps are taken to express these needs more confidently, and clients' ability to take more effective action on their own behalf develops. Eventually, bulimic behavior as a way of coping is outgrown.

The application of these principles in therapeutic practice will be demonstrated via a brief session excerpt.

